# Personalized Treatment of H3K27M-Mutant Pediatric Diffuse Gliomas Provides Improved Therapeutic Opportunities

**DOI:** 10.3389/fonc.2019.01436

**Published:** 2020-01-10

**Authors:** Johannes Gojo, Zdenek Pavelka, Danica Zapletalova, Maria T. Schmook, Lisa Mayr, Sibylle Madlener, Michal Kyr, Klara Vejmelkova, Martin Smrcka, Thomas Czech, Christian Dorfer, Jarmila Skotakova, Amedeo A. Azizi, Monika Chocholous, Dominik Reisinger, David Lastovicka, Dalibor Valik, Christine Haberler, Andreas Peyrl, Hana Noskova, Karol Pál, Marta Jezova, Renata Veselska, Sarka Kozakova, Ondrej Slaby, Irene Slavc, Jaroslav Sterba

**Affiliations:** ^1^Department of Pediatrics and Adolescent Medicine and Comprehensive Center for Pediatrics, Medical University of Vienna, Vienna, Austria; ^2^Comprehensive Cancer Center Vienna, Medical University of Vienna, Vienna, Austria; ^3^Department of Pediatric Oncology, University Hospital Brno and Faculty of Medicine, Masaryk University, Brno, Czechia; ^4^International Clinical Research Center, St. Anne's University Hospital, Brno, Czechia; ^5^Department of Biomedical Imaging and Image-Guided Therapy, Medical University of Vienna, Vienna, Austria; ^6^Department of Neurosurgery, University Hospital Brno and Faculty of Medicine, Masaryk University, Brno, Czechia; ^7^Department of Neurosurgery, Medical University of Vienna, Vienna, Austria; ^8^Department of Pediatric Radiology, University Hospital Brno and Faculty of Medicine, Masaryk University, Vienna, Czechia; ^9^Regional Centre for Applied Molecular Oncology, Masaryk Memorial Cancer Institute, Brno, Czechia; ^10^Institute of Neurology, Medical University of Vienna, Vienna, Austria; ^11^Laboratory of Tumor Biology, Department of Experimental Biology, School of Science, Masaryk University, Brno, Czechia; ^12^Central European Institute of Technology, Masaryk University, Brno, Czechia; ^13^Department of Pathology, Faculty Hospital Brno, Brno, Czechia

**Keywords:** diffuse midline glioma, H3K27M, pediatric oncology, precision medicine, comprehensive molecular profiling

## Abstract

Diffuse gliomas with K27M histone mutations (H3K27M glioma) are generally characterized by a fatal prognosis, particularly affecting the pediatric population. Based on the molecular heterogeneity observed in this tumor type, personalized treatment is considered to substantially improve therapeutic options. Therefore, clinical evidence for therapy, guided by comprehensive molecular profiling, is urgently required. In this study, we analyzed feasibility and clinical outcomes in a cohort of 12 H3K27M glioma cases treated at two centers. Patients were subjected to personalized treatment either at primary diagnosis or disease progression and received backbone therapy including focal irradiation. Molecular analyses included whole-exome sequencing of tumor and germline DNA, RNA-sequencing, and transcriptomic profiling. Patients were monitored with regular clinical as well as radiological follow-up. In one case, liquid biopsy of cerebrospinal fluid (CSF) was used. Analyses could be completed in 83% (10/12) and subsequent personalized treatment for one or more additional pharmacological therapies could be recommended in 90% (9/10). Personalized treatment included inhibition of the PI3K/AKT/mTOR pathway (3/9), MAPK signaling (2/9), immunotherapy (2/9), receptor tyrosine kinase inhibition (2/9), and retinoic receptor agonist (1/9). The overall response rate within the cohort was 78% (7/9) including one complete remission, three partial responses, and three stable diseases. Sustained responses lasting for 28 to 150 weeks were observed for cases with *PIK3CA* mutations treated with either miltefosine or everolimus and additional treatment with trametinib/dabrafenib in a case with *BRAFV600E* mutation. Immune checkpoint inhibitor treatment of a case with increased tumor mutational burden (TMB) resulted in complete remission lasting 40 weeks. Median time to progression was 29 weeks. Median overall survival (OS) in the personalized treatment cohort was 16.5 months. Last, we compared OS to a control cohort (*n* = 9) showing a median OS of 17.5 months. No significant difference between the cohorts could be detected, but long-term survivors (>2 years) were only present in the personalized treatment cohort. Taken together, we present the first evidence of clinical efficacy and an improved patient outcome through a personalized approach at least in selected cases of H3K27M glioma.

## Introduction

Central nervous system (CNS) tumors represent the most common solid malignancies in childhood and are the leading cause of cancer-related death in this age group (1). Diffuse midline gliomas (DMG) with histone H3 lysine27-to-methionine mutations (H3K27M glioma) represent a highly aggressive subtype of glioma, which predominantly arise in children and young adults (2–4). The overall prognosis of H3K27M glioma is poor, displaying median survival rates of approximately 9 to 11 months irrespective of the tumor localization (5–9). Based on its uniform fatal prognosis, the presence of *H3K27M* mutation has already been implemented into the new WHO classification as being diagnostic for high-grade gliomas (10). To date, focal irradiation therapy remains the mainstay of therapy for H3K27M glioma, resulting in improved overall survival rates (11). Although additional systemic therapy is generally considered as beneficial (7, 12), no therapy regimen has yet been shown to exert superior effects (11, 13–15). Consequently, novel, improved therapeutic strategies for H3K27M glioma are needed.

Since the discovery of the molecular basis of H3K27M glioma, we and others have intensively studied the underlying molecular biology (8, 16–19). Large international efforts have enabled molecular analysis of a substantial number of these rare tumors showing that H3K27M glioma also comprises biologically and genetically heterogeneous tumors (8, 19). These studies have resulted in the identification of additional oncogenic driver alterations in H3K27M glioma. Interestingly, these events include mutation of well-described oncogenic pathways including cell-/DNA-damage repair mechanisms (*TP53, PPM1D, ATM, ATRX*) and receptor tyrosine kinase signaling pathways (*ACVR1, FGFR1, PIK3CA, PIK3R1, BRAF)* (4, 8, 18). Many of these genomic alterations represent therapeutically actionable targets (8). Similarly, DNA copy number aberrations leading to amplifications of known oncogenes such as *PDGFRA, EGFR, CDK6, KIT, KDR*, and *MET* as well as deletion of tumor suppressors such as *CDKN2A* (8) denote equally appealing therapeutic targets. Additionally, we and others have shown that major driver alterations are present throughout the tumor tissue, suggesting that these trunc mutations are feasible therapeutic targets for the entire tumor bulk (19, 20). Moreover, the H3K27M protein has been proposed as promising neo-antigen making H3K27M gliomas potential candidates for immunotherapy (21).

Considering the fatal prognosis and the discovery of novel therapeutic targets in DMG, a variety of small clinical trials with novel targeted agents has already been conducted. Treatment with vinorelbine in combination with nimotuzumab, an antibody directed against *EGFR*, for example, has been shown to prolong survival, resulting in a median overall survival of 15 months (13). However, other studies with either EGFR-directed small molecules (gefitinib, erlotinib) or antibodies could not confirm this effect for all DMGs but showed individual cases of longer survival (7, 15, 22–24). Similarly, also therapy with dasatinib and crizotinib, two small-molecule PDGFRA inhibitors, has not shown overall survival benefit (25). Therefore, considering the aforementioned heterogeneity within H3K27M glioma, a “one-size-fits-all” approach does not appear to substantially improve patient outcome.

Comprehensive dissection of the molecular signatures and specific targeting of these molecular driver signals is hoped to significantly improve mortality and morbidity of cancer patients (26). Personalization of therapy is of particular interest in poor prognosis tumors such as H3K27M glioma and in tumors where inconsistent gene alterations exist (27). As pediatric tumors harbor much less mutations than do adult cancers, precision targeted therapy is likely to be more effective against these tumors than standard population-based approaches (27, 28). This has been corroborated by a recent prospective analysis confirming the presence of potentially targetable alterations in 76% of H3K27M-positive pontine gliomas (29). Additionally, a recently reported pilot study for DIPG has also reported feasibility of personalized treatment recommendations (30). Although multiple interventional molecular matching studies are ongoing (NCT01182350, NCT02233049), evidence for the clinical benefit of this approach in H3K27M glioma is still lacking.

Here, we investigated the feasibility and clinical benefit of comprehensive molecular profiling for H3K27M glioma in an international collaboration of two centers.

## Materials and Methods

### Case Selection

All prospectively evaluated patients aged 0–21 years with DMG diagnosed between 2015 and 2018 were retrospectively collected and included into the case series. Tumor biopsies yielding fresh tissue were performed at diagnosis as part of standard of care treatment. Confirmed histopathological diagnosis of high-grade glioma with H3K27M mutation was necessary for inclusion and further comprehensive molecular profiling. Informed consent was obtained from every participating patient and/or legal representative.

### Patient Treatment

All patients received backbone therapy consisting of focal irradiation and a systemic therapy backbone as by institutional guidelines (Table 1). Following comprehensive molecular profiling performed at CEITEC, Massaryk University Brno, patients were assigned to additional concomitant personalized treatment plans according to the consensus report of an interdisciplinary molecular tumor board. Respective treatment approaches were suggested according to previously described target actionability described in the INFORM trial (42), FDA datasheets, and Drugbank Canada (35), at mycancergenome.org, or described in other tumors, preclinical studies, or case reports as outlined in Table 1. If information on blood–brain barrier penetrance or effect in brain tumors was available from the literature, CNS-penetrant drugs were favored. Patient treatment with innovative therapeutics was based on named-patient use and informed consent was obtained from patients and/or legal representatives. Medication doses were chosen according to the literature and previous experience in the pediatric population if available.

**Table 1:** Clinical parameters, molecular alterations, line of treatment, treatment modalities, and backbone treatment of patients treated with personalized approaches.

**#**	**Center**	**Age**	**Gender**	**Localization**	**Molecular alteration**	**Line of treatment**	**Personalized treatment**	**Mode of action /rationale**	**Literature**	**Backbone treatment**	**OS (months)**
1	Brno	4.9	m	Pons	PIK3CA(E545K)	First	Miltefosin (2 mg/kg/day once daily)	AKT inhibitor	(31, 32)	RTX, nimotuzumab 150 mg/m^2^ + vinorelbine 20 mg/m^2^ every 7 days for 12 weeks, followed by nimotuzumab 150 mg/m^2^ + vinorelbine 25 mg/m^2^ every 14 days, valproate (plasma level 80–100 μg/ml)	44.5
2	Brno	4.9	f	Pons	ACVR1(R206H)	First	Palovarotene (0.4 mg/kg/day once daily)	Active in germline ACVR1 mutation	(33)	RTX, nimotuzumab 150 mg/m^2^ + vinorelbine 20 mg/m^2^ every 7 days for 12 weeks, followed by nimotuzumab 150 mg/m^2^ + vinorelbine 25 mg/m^2^ every 14 days, valproate (plasma level 80–100 μg/ml)	16.5
3	Brno	18.2	m	Pons	TMB 20 mut/MB	First	Nivolumab (1 mg/kg every 2 weeks first 4 months followed by 3 mg/kg every 2 weeks)	Immune checkpoint inhibitor	(34)	RTX, nimotuzumab 150 mg/m^2^ + vinorelbine 20 mg/m^2^ every 7 days for 12 weeks, followed by nimotuzumab 150 mg/m^2^ + vinorelbine 25 mg/m^2^ every 14 days, valproate (plasma level 80–100 μg/ml)	17.5^*^
4	Brno	6.4	f	Pons	PIK3CA(E545K)	First	Miltefosin (2.5 mg/kg/day once daily)	AKT inhibitor	(31, 32)	RTX, nimotuzumab/vinorelbine, valproate	15.0
7	Brno	6.6	f	Pons	FGFR3/CSF1R mRNA overexpression	Second	Pazopanib (5 mg/kg once daily, dose reduction due to side effects to 200 mg every other day)	Receptor tyrosine kinase inhibitor	Drugbank Canada (35)	RTX, nimotuzumab/vinorelbine, valproate	8.0
8	Brno	19.0	m	Spinal (lower thoracic region)	KRAS(G12A)	First	Trametinib (2 mg once daily)	MEK inhibitor	NCT03704688 (36)	RTX, nimotuzumab 150 mg/m^2^ + vinorelbine 20 mg/m^2^ every 7 days for 12 weeks, followed by nimotuzumab 150 mg/m^2^ + vinorelbine 25 mg/m^2^ every 14 days, valproate (plasma level 80–100 μg/ml), metoclopramide (0.4 mg/kg/day three times daily)	12.9
9	Vienna	8.2	m	Thalamic	BRAF(V600E)	Second	Dabrafenib (5 mg/kg/day divided twice daily), trametinib (0.04 mg/kg/day once daily), bevacizumab (10 mg/kg every 2 weeks)	BRAF/MEK inhibitors	(37–39)	Re-RTX, temozolomide (concomitant to RTX 75 mg/m^2^/day once daily)	28.8
10	Vienna	12.9	m	Pons	PIK3CA(G118D)	Second	Everolimus (4.5 mg/m^2^/day once daily, increased until trough level 5–15 ng/ml)	mTOR inhibitor	(40)	Temozolomide (200 mg/m^2^/day for 5 days at 28-day cycles), mebendazole 1500 mg/day three times daily	21.4
12	Vienna	4.9	m	Pons	PDGFRA(R841_I843delinsL) XPC(P334H)	First	Pazopanib (260 mg/m^2^/day once daily) pembrolizumab (2 mg/kg every 3 weeks)	PDGFRA inhibitor Immune checkpoint inhibitor	(34, 35, 41)	RTX, temozolomide (40 mg/m^2^/day once daily)	6.1

* *Alive with disease; OS, overall survival; TMB, tumor mutational burden*.

### Patient Data

Clinical data were obtained from patient charts available at the respective treating centers.

### Criteria for Response and Progression

Radiological response was assessed by experienced pediatric neuroradiologists using regular magnetic resonance imaging (at least every 3 months) according to modified RANO criteria (43).

### Survival Analysis

Patients with confirmed *H3K27M* mutation where comprehensive molecular profiling was not possible (*n* = 2) or without targetable alterations (*n* = 1) were included into the control group. Moreover, 6 patients with confirmed *H3K27M* mutation treated at the respective centers before comprehensive molecular profiling became available were included in the control group. All patients of the control group were treated according to institutional guidelines with focal radiotherapy and systemic chemotherapy (Table 2). Overall survival was defined as time between first diagnosis by imaging until death.

**Table 2:** Clinical parameters, histone mutation status, and treatment of cases in the control cohort.

**#**	**Center**	**Age (years)**	**Gender**	**Localization**	**H3 mutation**	**First-line treatment**	**Second-line treatment**	**OS (months)**
5	Brno	4.9	f	Pons	IHC	Nimotuzumab/vinorelbine		19.0
6	Brno	8.2	m	Pons	IHC	Nimotuzumab/vinorelbine	Re-RTX	15.0
11	Vienna	5.9	f	Pons	H3F3A	Temozolomide	Re-RTX, everolimus	19.7
13	Vienna	8.8	m	Pons, mesencephalon	H3F3A	Tumor vaccination	Immune checkpoint inhibitors	10.7
14	Vienna	2.4	m	Pons	HIST1H3B	Temozolomide, tumor vaccination	Re-RTX	20.4
15	Vienna	8.4	m	Pons	H3F3A	Temozolomide		16.8
16	Vienna	9.8	f	Thalamus	H3F3A	Temozolomide	Intrathecal VP-16, PEI	7.9
17	Vienna	11.1	m	Pons, cerebellum	IHC	Nimotuzumab/vinorelbine	Re-RTX, PEI	19.4
18	Vienna	4.4	m	Pons, mesencephalon	IHC	Nimotuzumab/vinorelbine	PEI	17.8

*OS, overall survival; IHC, immunohistochemistry*.

### Whole Exome Sequencing

DNA was extracted from FFPE tumor tissue samples using the QIAmp DNA FFPE Tissue Kit (Qiagen, Netherlands). The whole exome libraries were prepared using TruSeqExome Kit (Illumina, CA, USA) according to the manufacturer's recommendations. Quantity and quality of exome libraries were checked using Qubit 2.0 Fluorometer and NanoDrop2000c spectrophotometer (Thermo Fisher Scientific). Prepared libraries were loaded onto NextSeq 500/550 Mid Output Kit (150 cycles) and sequenced on the NextSeq 500 instrument (both Illumina). Sequencing coverage for both exomes was >20× at >90% of capture regions.

### Bioinformatic Analysis

Sequencing reads in fastq format were mapped to the human reference genome GRCh37 with the bwamem algorithm for both the tumor and the healthy control sample. The resulting alignments in “bam” format were postprocessed with the samblaster program for marking PCR duplicates. The final alignment file of the control sample was used to assess single nucleotide variants (SNVs) and short insertions/deletions (indels). Two variant callers were used for germline variant calling; the GATK HaplotypeCaller and VarDict (AstraZeneca, Waltham, MA, USA). Reported variants were annotated with Annovar and Oncotator annotation programs. Tumor-specific variants were assessed by somatic (paired; tumor vs. control) variant calling. For this purpose, we used Mutect (SNVs), Scalpel (Indels), and VarDict (SNVs and Indels) variant callers. The annotation of somatic variants was performed with the addition of the COSMIC database.

Variants were filtered manually based on the virtual panel filtering (genes analyzed by FoundationOne CDx panel, genes that are cataloged in the Cancer Gene Census, and variants that have previously been reported in COSMIC, MD Anderson). Mutations in genes that have been causally implicated in cancer are then manually checked in other available databases or scientific literature sources (e.g., cBioPortal, The Clinical Knowledgebase—JAX CKB, MyCancerGenome) where their potential oncogenic biological effect and references to relevant clinical trials or studies can be found. Selected gene variants with known or potential clinical significance are outlined in the final report; other variants found are listed separately (Table S1) and are considered as variants of uncertain clinical significance (VUS).

### Tumor Mutational Burden Estimation

An annotated list of somatic variants from the previous step is used to assess the tumor mutation burden (TMB). For TMB calculation from WES data only somatic point mutations were considered, since indels (short insertions and deletions) tend to be called with high false-positive rates and could potentially skew the outcome. Additionally, two bases before and after each exon are considered for splicing mutations. Synonymous variants are filtered out, as they do not fit the definition of TMB. Finally, variants with variant allele frequency of <5% are filtered out. The coding region locations on the hg19 genome were downloaded from the UCSC genome browser.

### Detection of Fusion Genes by Next-Generation Sequencing

Total RNA from tumor tissue was extracted using mirVana miRNA Isolation Kit (Thermo Fisher Scientific, MA, USA). Quantity and quality of extracted RNA were checked by Qubit® 2.0 Fluorometer system (Thermo Fisher Scientific, MA, USA) and NanoDrop 2000c Spectrophotometer (Thermo Fisher Scientific, MA, USA). For sequencing libraries preparation, TruSight RNA Pan-Cancer Panel (Illumina, CA, USA), which targets fusions in 1385 genes, was used. Sequencing libraries were subsequently loaded on NextSeq 500/550 Mid Output Kit v2 (150 cycles) and NextSeq 500 sequencing device (both Illumina, CA, USA). All processes were performed according to the manufacturer's instructions. Quantity and quality of sequencing libraries were checked by Qubit® 2.0 Fluorometer system (Thermo Fisher Scientific, MA, USA) and TapeStation 2200 (Agilent Technologies, CA, USA). For data analysis, BreakingPoint tool was used.

### Liquid Biopsy Analysis of Cerebrospinal Fluid (CSF)

CSF was obtained via lumbar puncture at the given time points. The cfDNA isolation from 1 ml CSF was performed using the quick cfDNA/cfRNA serum and plasma kit (Zymo Research, CA, USA) following the manufacturer's instructions. The QX200^TM^ digital droplet system from BioRad (CA, USA) was used and the assay was performed according to manufacturer's manuals. In brief, the unique assay ID dHsaMDV2510510 for the *H3F3A* p.K28M mutation from BioRad was used to analyze the mutations in the cfDNA of patient CSF samples. To each run, a sample with known positive H3F3A p.K28M mutation and a negative control (nuclease free water) were included to determine the fluorescence thresholds. The results of ddPCR were analyzed with Quantasoft^TM^ software. Detected counts of *H3F3A* mutant and wild-type cfDNA were normalized to 1 ml CSF volume. Thereby, samples of different time points could be compared for semiquantitative longitudinal analysis.

### Statistical Analysis

Analyses were performed using SPSS version 25.0 and Graph Pad Prism version 5.0.

## Results

### Study Cohort and Comprehensive Molecular Profiling

**Figure 1:**
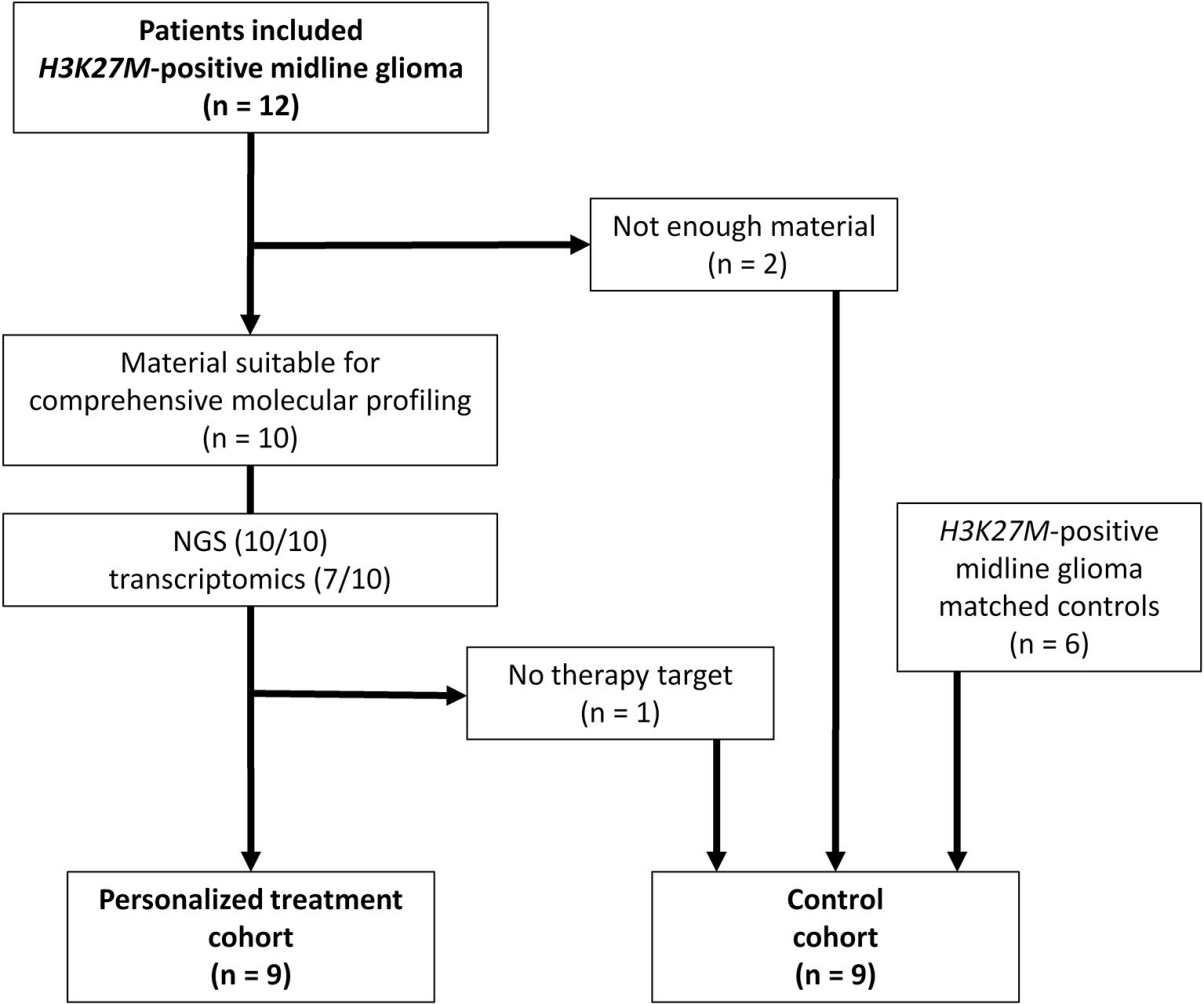
Flow chart of patients included in personalized treatment and control cohort. NGS, next-generation sequencing.

**Figure 2:**
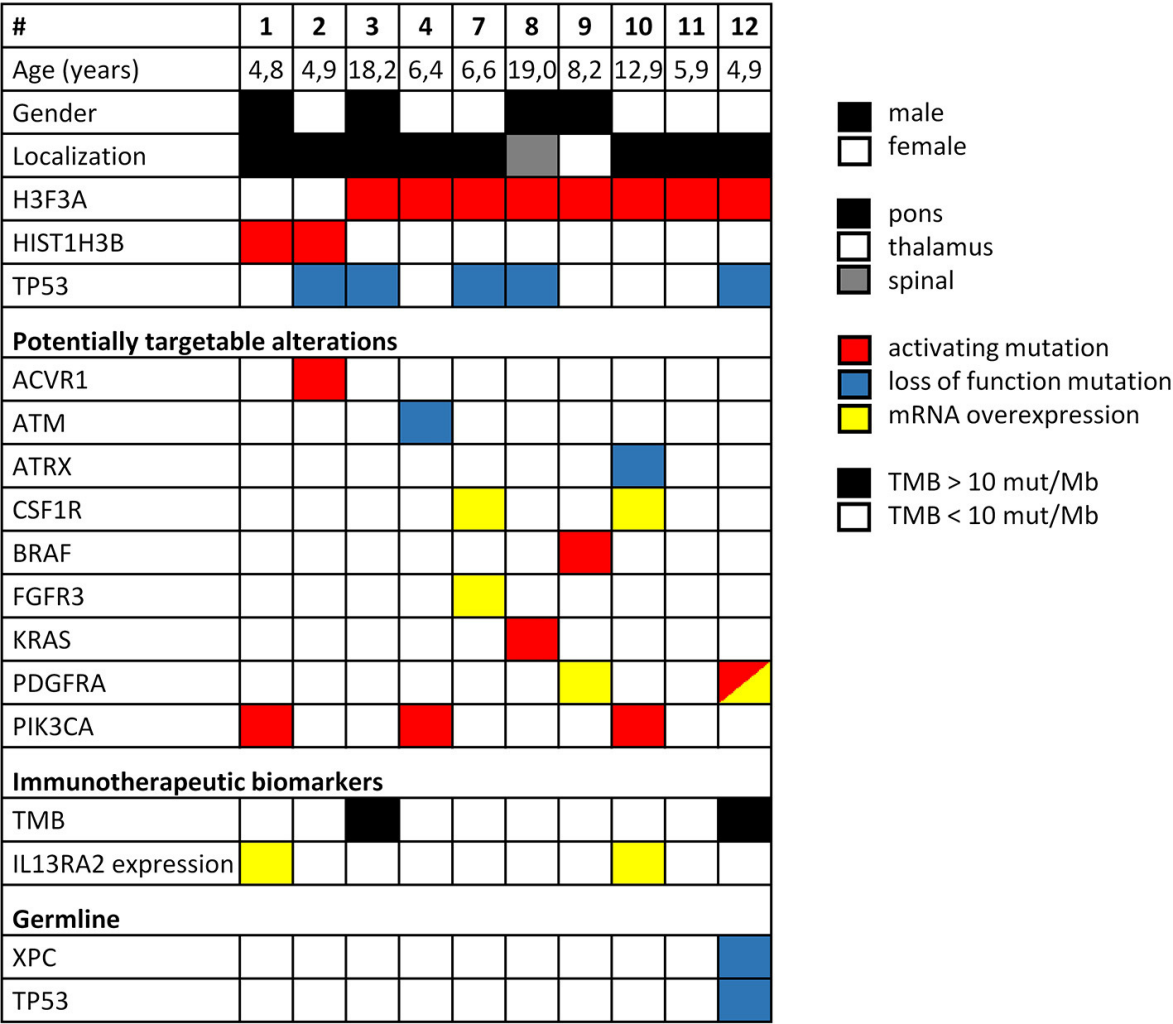
Overview of clinical parameters and detected molecular alterations for H3K27M glioma analyzed by comprehensive molecular profiling. TMB, tumor mutational burden.

Twelve patients were included in this study whereby two cases had to be excluded due to insufficient amount of biological material available (Figure 1). Comprehensive molecular profiling was performed for 10 tumors (Figure 1). Next-generation sequencing could be performed for all 10 cases and transcriptomic profiling in 7 out of 10. *H3K27M* mutations were confirmed in all cases (*HIST1H3B* 2/10, *H3F3A* 8/10). Mutations in *TP53* were detected in 5 of the tumors analyzed (5/10). Potentially targetable mutations included *KRAS* (1/10), *PIK3CA* (3/10), *BRAF* (1/10), *ACVR1* (1/10), *ATM* (1/10), and *ATRX* (1/10) (Figure 2). With respect to possible immune checkpoint inhibitor therapy, high TMB (2/10) and overexpression of *IL13RA2* (2/13) were detected. One case was assigned to treatment solely based on transcriptomic profiling due to the lack of targetable mutations (case #7). Only one case (case #11) could not be assigned to a personalized treatment approach. Table 1 lists clinical details, detected molecular alterations, and personalized as well as backbone treatment for the respective cases. Alteration of the PI3K/AKT/mTOR pathway via mutation of *PIK3CA* was detected in three cases. The two cases harboring a *PIK3CA(E545K)* mutation were subsequently treated with miltefosine, an AKT inhibitor (31, 32), the one case with *PIK3CA(G118D)* mutation with everolimus, a, mTOR inhibitor approved for treatment of tuberosis sclerosis associated tumors (40). Based on effective treatment of germline *ACVR1* mutations with palovarotene, a retinoic receptor agonist, in the literature (33), the case with *ACVR1(R206H)* was treated accordingly. With respect to alterations of the MAPK pathway, one case with *BRAF(V600E)* mutation was subjected to treatment with a combination of dabrafenib, trametinib, together with bevacizumab, and the patient harboring a tumor with *KRAS(G12A)* was subjected to treatment with trametinib (36–39). Two patients (cases #3 and #12) were assigned to receive immunotherapeutic approaches due to high tumor mutational burden (TMB) (34). In one case (case #12), we additionally detected a germline mutation of *XPC*, which has been described as being susceptible toward immune checkpoint inhibition (41). The latter patient was additionally treated with pazopanib, targeting detected mutation and overexpression of *PDGFRA* (35). Last, another patient (case #7) was assigned to treatment with pazopanib based on detected mRNA overexpression of *FGFR3* and *CSF1R* (35).

### Response to Personalized Treatment

**Figure 3:**
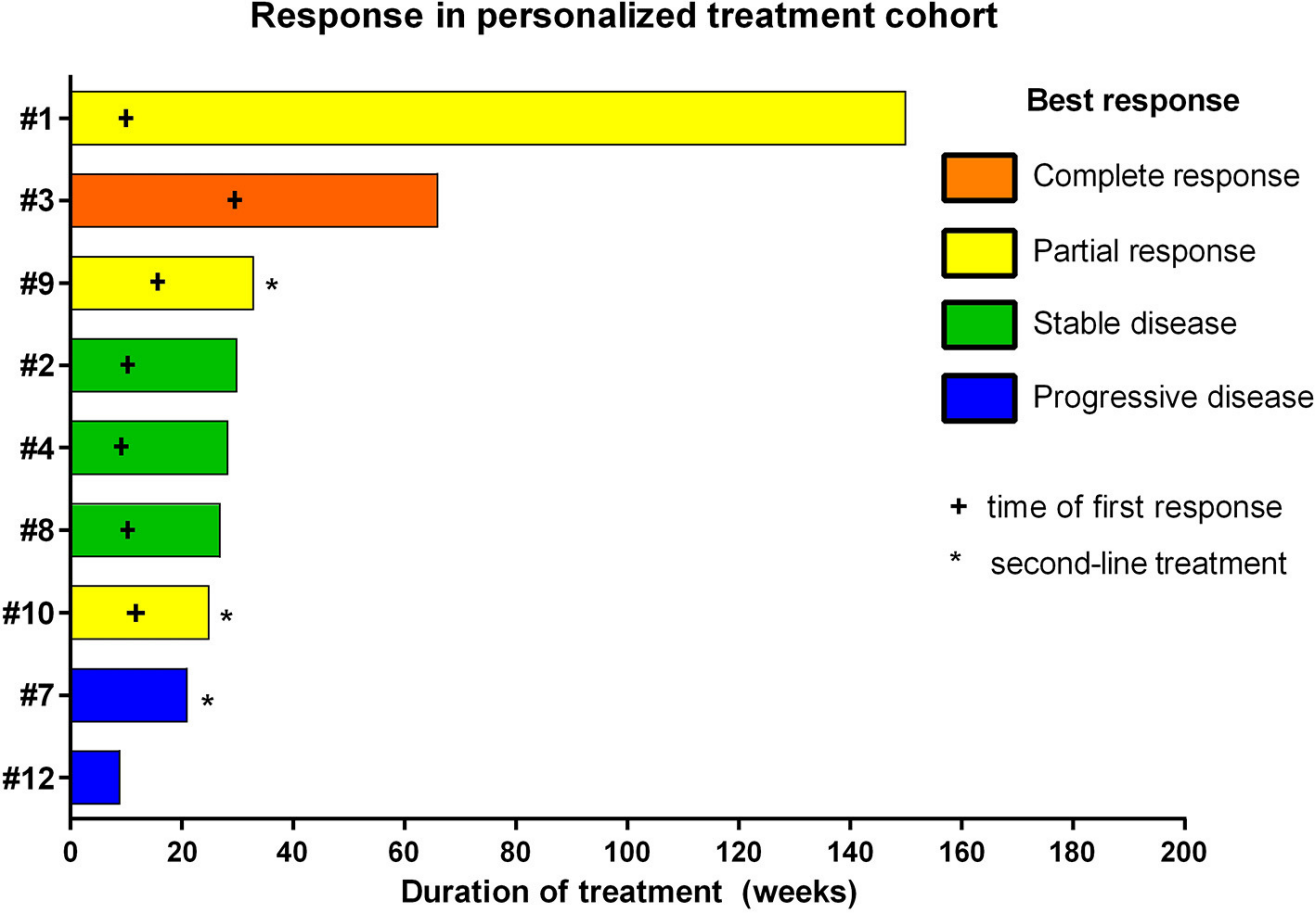
Individual responses of patients treated with personalized treatment approaches based on comprehensive molecular profiling.

The overall response rate was 78% (7/9), consisting of two stable diseases, three partial responses, and one complete response (Figure 3). Median time to progression was 25 weeks. It is worth noting that the analysis also included three patients who were treated with personalized approaches at first progression as second-line treatment and not upfront. The two responders of this group showed a progression-free survival of 25 and 33 weeks, respectively. The six cases treated upfront with molecularly guided treatment plans exhibited a median time to progression of 29 weeks. Two patients showed disease progression under personalized treatment approaches. Interestingly, both patients not responding to treatment were treated with pazopanib (cases # 7 and #12).

**Figure 4:**
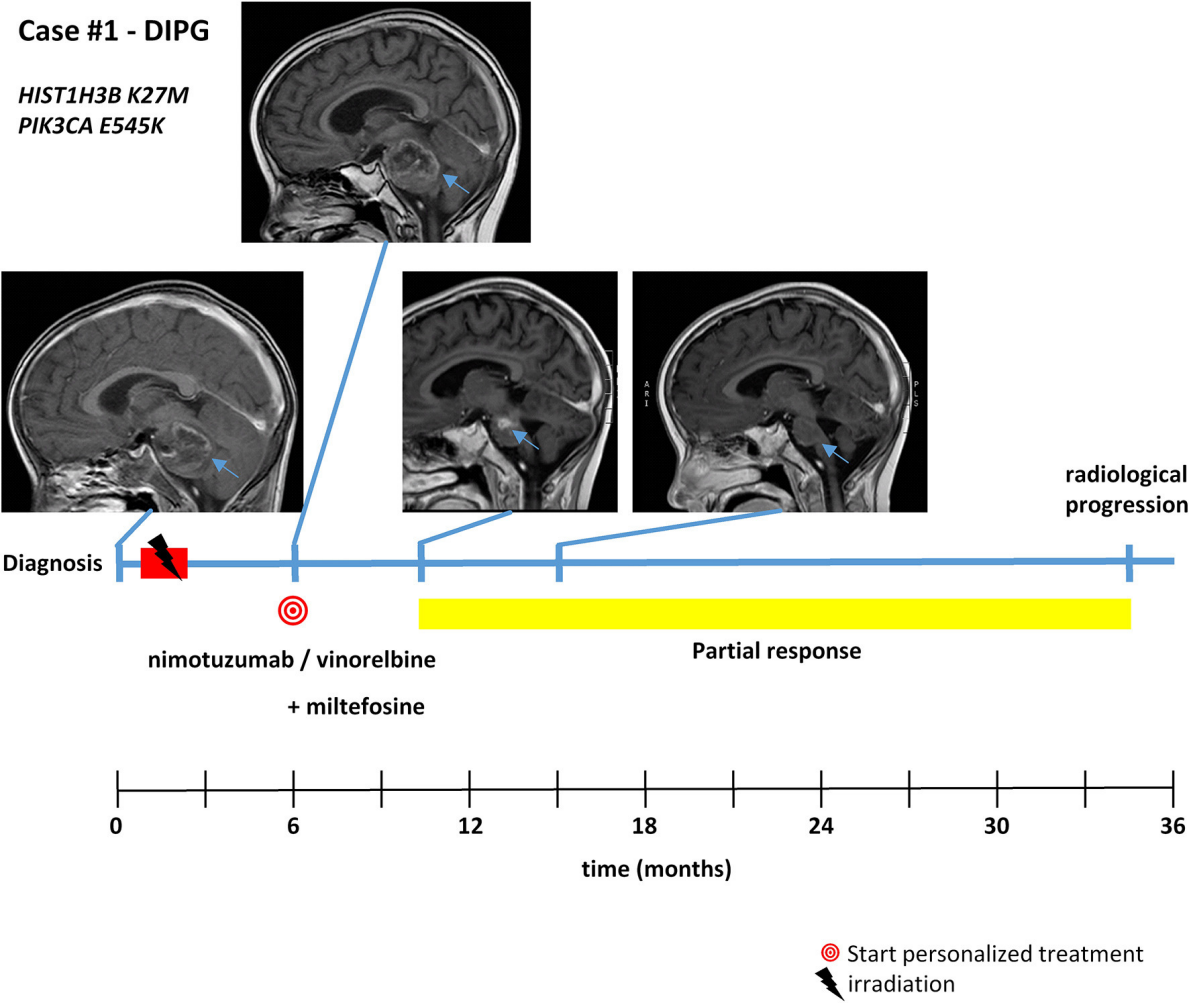
Detailed case description—case #1. Timeline of patient treatment including magnetic resonance images at indicated time points. Blue arrows indicate tumor. DIPG, diffuse intrinsic pontine glioma.

**Figure 5:**
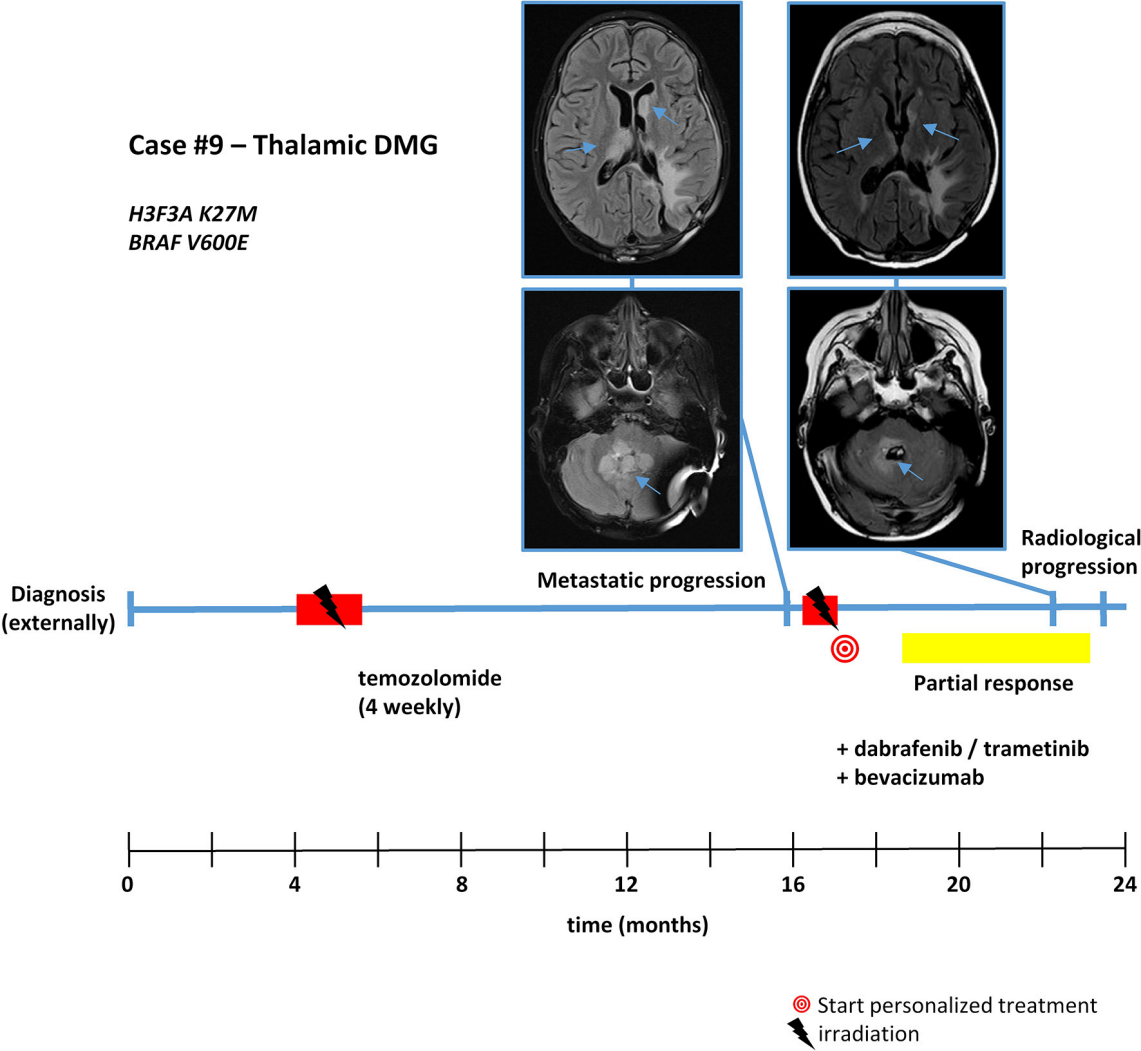
Detailed case description—case #9. Timeline of patient treatment including magnetic resonance images at indicated time points. Blue arrows indicate tumor. DMG, diffuse midline glioma.

**Figure 6:**
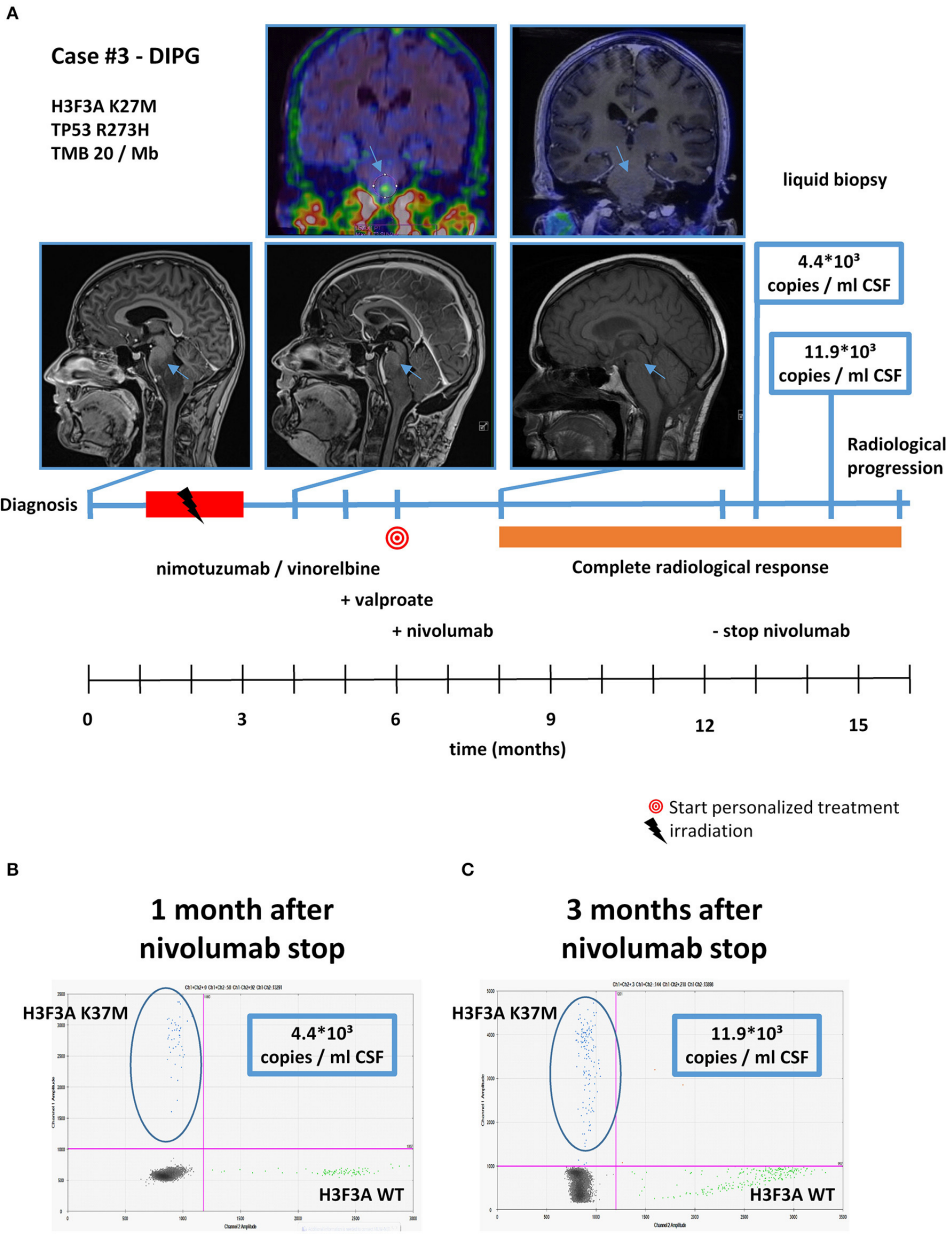
Detailed case description—case #3. **(A)** Timeline of patient treatment including magnetic resonance images, ^18^F-FET-PET images, and liquid biopsy results at indicated time points. Blue arrows indicate tumor. **(B)** Digital droplet PCR (ddPCR) plot in cerebrospinal fluid (CSF) 1 month after discontinuation of nivolumab. **(C)** ddPCR plate in CSF 3 months after discontinuation of nivolumab. DIPG, diffuse intrinsic pontine glioma.

With respect to molecular alterations, miltefosine treatment of tumors harboring *PIK3CA(E545K)* resulted in one stable disease (case #4) and one partial response (case #1, Table 1, Figure 3). In the latter case, the patient was treated with miltefosine, an AKT inhibitor, in addition to nimotuzumab and vinorelbine following irradiation (case #1, Figure 4), resulting in a prolonged partial response of 150 weeks. Everolimus treatment in the case with *PIK3CA(G118D)* led to partial response in second-line treatment (case #10, Table 1, Figure 3). In the case of a thalamic tumor harboring an additional *BRAF(V600E)* mutation, comprehensive molecular profiling was performed at the time of progression where multiple metastatic lesions were detected (case #9, Figure 5). Personalized treatment following re-irradiation in addition to a temozolomide backbone resulted in shrinkage of the lesions and partial response even in second-line treatment. Also in the case of a *KRAS(G12A)* mutation, treatment with trametinib in addition to irradiation, nimotuzumab, vinorelbine, and metoclopramide resulted in stable disease (case #8, Table 1, Figure 3). Moreover, also personalized treatment of *ACVR1* mutation with palovarotene (case #2, Table 1, Figure 3) resulted in disease stabilization, providing first evidence of this approach for *H3K27M-ACRV1* commutated tumors. Finally, adding immune checkpoint inhibition to the backbone treatment based on high TMB resulted in a sustained complete remission in one case (case #3, Figure 6A). However, after 8 months of treatment, the patient developed severe autoimmune encephalitis necessitating treatment interruption. During steroid treatment, the patient improved markedly. Additionally performed liquid biopsy analysis for *H3K27M* in CSF documented an increase of the *H3K27M* copies during treatment gap, followed by radiological and clinical progression later on (Figures 6A,B). The second case treated with immune checkpoint inhibition (case #12, Table 1, Figure 3) showed no response despite high TMB (27/MB) resulting from a germline *XPC* mutation. It has to be noted, however, that this case displayed an extraordinary aggressive phenotype with massive clinical and radiological progression in only 10 days prior to biopsy and treatment start. Moreover, immune checkpoint inhibitors could only be introduced after the disease had already progressed despite radiotherapy and the patient only received four cycles.

### Survival Analysis

**Figure 7:**
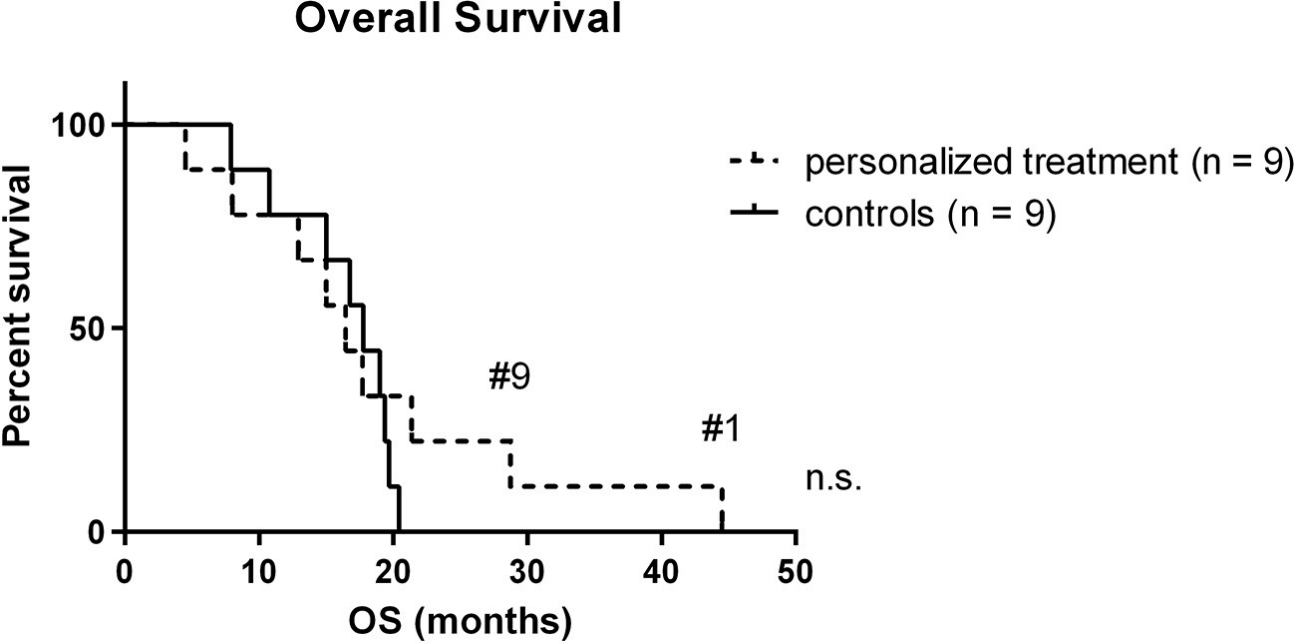
Kaplan-Meier plot of personalized treatment and control cohort. Long-term survivors are indicated. OS, overall survival.

**Figure 8:**
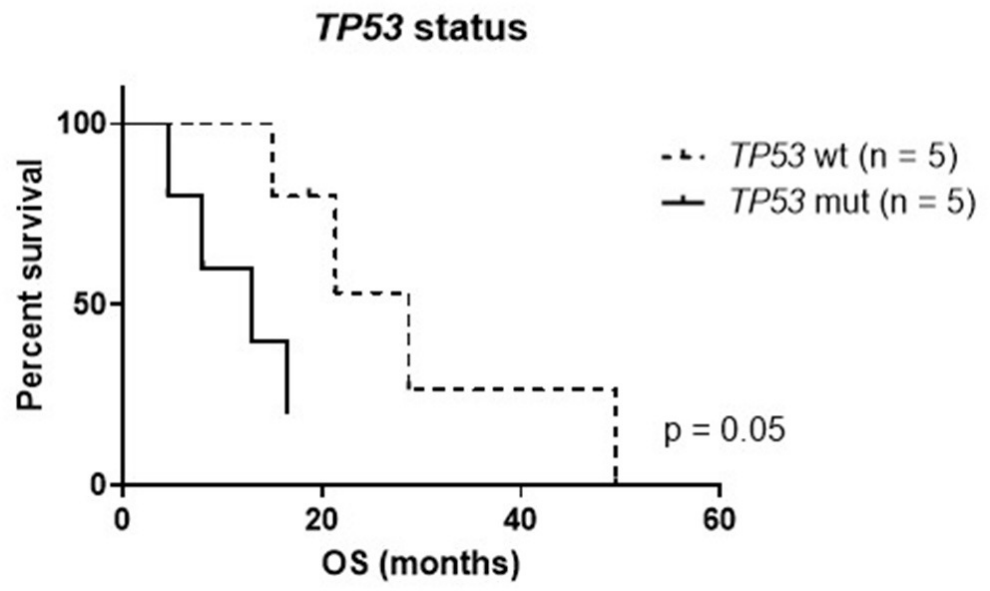
Kaplan–Meier plot of *TP53* mutant (mut) and wild-type (wt) cases within the personalized treatment cohort. OS, overall survival.

To evaluate the potential impact of personalized treatment approaches on the overall survival of H3K27M glioma patients, we set up a control cohort of patients treated at the same centers for comparison. The clinical details of the control cohort are outlined in Table 2. Comparison of clinical parameters in the personalized and the control cohort is given in Table 3. At the time of data analysis (July 2019), no patient in both the control and the personalized treatment cohort remained alive. Median overall survival was 16.5 months (12.2–20.8 months 95% CI) in the personalized and 17.8 months (14.8–20.7 months 95% CI) in the control cohort, respectively (Figure 7). The hazard ratio for personalized treatment was 0.69 (0.25–1.95 95% CI). Accordingly, no significant difference between the two groups was observed. 1-year OS in both cohorts was 77% (±14%). In contrast, 2-year OS was 11% (±10%) in the personalized treatment cohort, whereas no patient in the control cohort survived longer than 2 years. The longest observed survival was 44.5 months. As *TP53* mutation was the second most common recurrent aberration in our case series, we compared survival rates of *TP53* wild-type and mutant cases (Figure 8). *TP53* mutant cases showed a markedly shorter overall survival (median survival 12.9 months) as compared to *TP53* wild-type cases (median OS 28.7 months).

**Table 3:** Comparison of clinical and H3K27M-status in personalized and control cohort.

	**Personalized**	**Control**
Age in years, median (range)	6.6 (4.8–19)	8.2 (2.4–11.1)
Gender (m:f)	4:5	6:3
**Localization**		
Pons	7	8
Thalamus	1	1
Spinal	1	–
**H3K27M detection**		
H3F3A	7	4
HISTH3B	2	1
IHC	0	4
**Backbone treatment (first line)**		
Nimotuzumab/vinorelbine	6	4
Temozolomide	3	4
Other	0	1

*IHC, immunohistochemistry*.

## Discussion

Due to the uniformly fatal prognosis of H3K27M glioma, improved therapeutic strategies are urgently needed. Recent high-throughput studies led to the conclusion that despite shared H3K27M mutation, these entity comprises multiple different molecular subgroups (8, 19). Being controversially discussed some years ago, biopsy of DMGs has been shown to be safe, which we could also confirm in our case series (29, 30). Hence, personalized treatment approaches based on comprehensive molecular profiling are considered a particularly promising treatment approach (27). In the underlying study, we investigated not only the presence of potential treatment targets but also clinical benefit via individualized treatment plans. To our knowledge, this is the first personalized medicine-based study describing response rates in H3K27M glioma. Thus, despite low patient number and non-prospective design of the study, we consider these results of high interest to the medical community.

Several previous studies assessed the potential of molecular approaches in H3K27M glioma (4, 8, 18, 29); however, data on the clinical impact is limited (30). In contrast to a recently published study in DIPG (30) our study cohort was restricted to H3K27M glioma (8/10 H3F3A, 2/10 HIST1H3B), also including a thalamic and a spinal case. Comparable to previous studies, 50% of tumors harbored a mutation in *TP53* (29, 30). By comprehensive molecular profiling, we detected targetable alterations in 90% (9/10) of the tumors, which is comparable to previous results from the INFORM study. In contrast to the aforementioned study in DIPG, targeting H3K27M glioma with HDAC inhibitors such as panobinostat was not considered “targeted treatment recommendation” in our study cohort (30). Valproate was used in 6/9 cases as therapy backbone. There were no two molecularly identical patients in our cohort, suggesting the importance of a personalized approach even within a relatively narrow and already molecularly predefined group like H3K27M glioma. While most tumor cells harbor more than one tumor-propagating change within the different cell-signaling pathways, progressive or relapsed tumors display additional molecular changes mediating resistance to previous treatments as we could document in our serial samples as well (data not shown). It remains a great challenge to address more than one or two such aberrations, and such combination treatments should be based on strong scientific rationale.

In our personalized treatment cohort, the overall response rate was 78% (7/9). Interestingly, the two non-responders were both treated with pazopanib, suggesting poor benefit from pazopanib treatment for H3K27M glioma. However, both tumors also harbored a *TP53* mutation, which we show to be associated with an inferior outcome.

One third of the cases (3/9) exhibited activation of the PI3K/AKT/mTOR pathway that was targeted by either miltefosine or everolimus. All patients responded to a combination with backbone treatment, which also included the case with the longest observed OS (44.5 months) in this cohort. Consequently, our data indicate that targeting molecular alterations of the PI3K/AKT/mTOR pathway in H3K27M glioma might represent a promising therapeutic approach worth validating in clinical trials that have already been initiated (27).

One thalamic case harbored an additional *BRAF(V600E)* mutation. This co-occurrence has already been described and diffusely infiltrating tumors in the midline appear to carry the same dismal prognosis as other H3K27M gliomas (18, 30, 44). However, patients with double-mutant tumors may also show a more benign course of disease, in particular if they show low infiltration, low-grade glioma histology features and can be safely resected (45). In contrast, the case in our cohort showed high proliferation and was treated at metastatic disease progression with a combination of trametinib, dabrafenib, and bevacizumab in addition to backbone treatment. We observed a partial remission lasting 33 weeks. OS in this case was 28.8 months, suggesting a benefit of targeted treatment in *BRAF* commutated H3K27M glioma. The second case treated with the MEK inhibitor trametinib harbored a *KRAS(G12A)* mutation and also showed stable disease lasting 28 weeks.

Activating mutations in *ACVR1* have been reported in approximately 20% of DIPG (8, 46). Interestingly, previous reports in patients with germline *ACVR1* mutations suggested benefit from treatment with palovarotene, a retinoic receptor agonist (33). The single case with *ACVR1* mutation in our cohort was treated accordingly, resulting in disease stabilization lasting 30 weeks.

Immunotherapy has revolutionized oncology in the past years and resulted in substantial improvement of treatment outcomes in certain tumor types (47). In pediatric high-grade glioma, best outcomes were described for patients harboring germline mutations in DNA repair mechanisms (34). Within our cohort two patients with increased TMB were treated with immune checkpoint inhibitors. One of these patients showed the sole complete remission in the whole cohort. However, treatment had to be discontinued due to severe side effects, and during steroid treatment, rapid disease progression was observed. In the second case with high TMB due to a germline *XPC* mutation, immune checkpoint inhibitor treatment in addition to pazopanib was recommended. In this case, we observed a highly aggressive course of disease, already prior to personalized treatment approaches and no response under immune checkpoint inhibition. Consequently, immune checkpoint inhibition appears to be a highly effective treatment in selected cases but testing for tumor mutational load appears to be crucial to predict benefit of treatment. However, for future trials, investigation of tumor microenvironment and immune response appear to be crucial in order to further clarify which patient collective may benefit from immune checkpoint inhibitors. Moreover, in our series, immunotherapies were applied following radiotherapy. Interestingly, recent analyses in adult high-grade glioma suggest that immunotherapies may be more effective if applied before irradiation (48). In our longest surviving patient (case #1), immunotherapy was added only after the disease progression as second personalized treatment approach, 35 months from original diagnosis. Therapy consisted of four doses of autologous dendritic cell vaccine according to our institutional protocol (EudraCT number 2014-003388-39). Accelerated disease progression was documented after corticosteroid treatment being a component of terminal antiedematous approach. These observations may stimulate further studies addressing immunotherapeutic approaches for this particularly fatal malignancy.

As the number of oncogenic mutations is generally low in H3K27M glioma, we also included transcriptomic profiling in addition to mutation detection in 70% of the cases. Treatment recommendation was solely based on transcriptomic profiling in one case with elevated *FGFR3* and *CSF1R* expression. Treatment with the receptor tyrosine kinase inhibitor pazopanib did not show an effect. Despite this discouraging observation, we suggest to include transcriptomic profiling also in future personalized medicine approaches as some important aspects like immune evasion or angiogenesis are not reflected just by mutational analyses. The poor effect may have been based on the lack of clinical efficiency with pazopanib treatment, which has also been described for adult high-grade glioma (49). With respect to the emerging treatment with ONC201 (50), being currently assessed within trials, we retrospectively analyzed *DRD2* expression. In all our investigated cases, the *DRD2* expression was decreased (data not shown); however, recent data are suggesting different mechanisms of action for ONC201 (51).

Overall analysis of the clinical benefit demonstrated a median PFS of 29 weeks, also including second-line treatment cases. This might also be the reason for a shorter PFS as compared to other first-line studies (13, 22, 23) but longer median PFS as compared to a previous study for recurrent tumors (24). To assess potential benefit for overall survival of a personalized treatment approach in H3K27M glioma, we compared the cohort to a retrospective control cohort treated at the same centers. No significant difference between the two cohorts was observed (16.5 vs. 17.8 months). It must be noted that in both cohorts, the median OS was markedly higher than in recent meta-analyses reporting an OS of <1 year (7, 9). For example, a recent large retrospective analysis reported a median OS of 10.4 months for *H3F3A*-mutated and 15.0 months for *HIST1H3B* (7). A median OS of 15 months was reported in a study investigating the combination of nimotuzumab and vinorelbine. This backbone was used in 6/9 cases in personalized and in 4/9 cases in the control cohort of our study. Interestingly, median OS for both of the cohorts was longer. Consequently, no significant benefit of personalized treatment approaches was observed, also owing to the good survival rates within the control group. However, longer survival beyond 2 years was only seen in the personalized treatment cohort. We are aware that the study design and results do not allow a clear conclusion whether personalized treatment is of benefit in H3K27M glioma. However, the molecular profiles of the analyzed cases reveal that we did not find any identical case. Consequently, a randomized study approach is less attractive given the strong molecular heterogeneity within this tumor type. Nevertheless, a prospective trial with a larger sample size would be urgently needed to further assess the potential of personalized treatment approaches in this devastating disease.

Liquid biopsy has emerged as a promising diagnostic tool for improved patient monitoring, also in H3K27M glioma (30, 52). The utility in real-world application, however, has not yet been widely investigated. Herein, we report an increase of H3K27M copy numbers in CSF of a patient in complete radiological remission 3 months prior to detection of radiological progression. This underlines the opportunities for tumor DNA detection in CSF for future therapy guidance in H3K27M glioma patients. However, these methods need further validation in larger patient cohorts before they can be routinely applied for assessment of treatment response or recurrence.

Taken together, we show that personalized treatment approaches that address molecular heterogeneity of H3K27M glioma based on tumor biopsies are safe and feasible. Moreover, we demonstrate that clinical efficacy in selected cases is worth validating in future clinical trials with larger patient numbers.

## Data Availability Statement

The datasets generated for this study are available on request to the corresponding author.

## Ethics Statement

This study was approved by the local ethics committee of the Medical University of Vienna and the Masaryk University Brno. Informed consent was obtained from every participating patient and/or legal representative.

## Author Contributions

JG, JSt, and IS designed the study, interpreted data, and wrote the manuscript. JG, ZP, DZ, LM, MK, KV, MS, TC, CD, AA, MC, DR, DL, AP, JSt, and IS acquired clinical data, were involved in molecular tumor board decisions, and managed patients. MTS and JSk performed radiological assessments. CH, DV, HN, KP, OS, MJ, RV, and SK performed histopathological evaluation and molecular analyses for comprehensive molecular profiling. SM performed liquid biopsy analyses.

### Conflict of Interest

The authors declare that the research was conducted in the absence of any commercial or financial relationships that could be construed as a potential conflict of interest.

## Supplementary Material

Table S1 Whole exome sequencing results.
